# Schistosomiasis Haematobium, Corsica, France

**DOI:** 10.3201/eid2009.140928

**Published:** 2014-09

**Authors:** Antoine Berry, Hélène Moné, Xavier Iriart, Gabriel Mouahid, Olivier Aboo, Jérôme Boissier, Judith Fillaux, Sophie Cassaing, Cécile Debuisson, Alexis Valentin, Guillaume Mitta, André Théron, Jean-François Magnaval

**Affiliations:** Centre Hospitalier Universitaire, Toulouse, France (A. Berry, X. Iriart, O. Aboo, J. Fillaux, S. Cassaing, C. Debuisson, A. Valentin);; Institut National de la Santé et de la Recherche Médicale, Toulouse (A. Berry, X. Iriart);; Centre National de la Recherche Scientifique, Perpignan, France (H. Moné, J. Boissier, G. Mouahid, G. Mitta, A. Théron);; Centre National de la Recherche Scientifique, Toulouse (J.-F. Magnaval)

**Keywords:** Schistosoma haematobium, schistosomiasis haematobium, schistosomiasis autochthonous transmission, Bulinus truncatus, snails, reemergence, Europe, Corsica, France

**To the Editor:** In Europe, urinary schistosomiasis (*1*) has previously been detected only in Portugal, where this focus disappeared during the 1950s (*2*). However, freshwater snails of the species *Bulinus contortus*, *B*. *truncatus*, and *Planorbarius metidjensis*, which are recognized intermediate hosts for *Schistosoma haematobium* trematodes, have been found in Portugal (*3*), Spain (*4*), and Corsica (*5**,**6*). This finding suggested that autochthonous schistosomiasis could re-emerge in southern Europe if these mollusks become infected. We report a probable focus for transmission of schistosomiasis haematobium in Corsica, France.

In March 2014, a 4-year-old girl (index case-patient) from France was referred to the Toulouse University Hospital (Toulouse, France), with gross hematuria. Ultrasonography and cystoscopic examination of the bladder detected a polyp. Examination of the polyp for parasites identified bodies that were consistent with schistosome eggs. Parasitologic examination of urine confirmed schistosomiasis by detecting viable *S. haematobium* eggs.

The parents of the girl (family A) did not report any stay or travel in an area to which urinary schistosomiasis was endemic; they reported summer holidays only in Mallorca in the Balearic Islands (Spain) and Corsica. However, her father reported that since 2012, he had experienced gross hematuria that had been evaluated by standard urologic investigations but not by cystoscopy; no etiology was determined. Parasitologic urinalysis in our hospital department showed numerous viable *S. haematobium* eggs in the father’s urine.

The parents of the index case-patient also reported that an 8-year-old boy in a friend’s family (family B), who shared summer vacations with them had exhibited gross hematuria since February 2013. A third family (family C) was also investigated because they also spent holidays in Corsica with families A and B. Families B and C had also spent a summer in Mallorca, but they denied any contact with freshwater. Of 11 French native-born members of the 3 families, 6 had positive results for *S. haematobium* by urine examination. All case-patients had specific positive immunodiagnostic results by an ELISA that used *S. mansoni* extracts and by indirect hemagglutination. In addition, 2 family members who had a negative result by urine examination had a positive serologic result.

Spending summer vacations in the same village in Corsica (Sainte-Lucie de Porto-Vecchio), where members of the 3 families had bathed at least once per holiday period in the Cavu River, was the epidemiologically prominent feature that linked these persons. Families A and C were in Sainte-Lucie de Porto-Vecchio in August 2011, and families A, B, and C were in the same location in August 2013.

During these investigations, we were contacted by the Department of Tropical Medicine, Dusseldorf University Hospital (Dusseldorf, Germany), because a 10-year-old boy and his father had been given diagnoses of schistosomiasis haematobium on the basis of positive urinalysis results for *S. haematobium* eggs. Two other members of this family (5 persons) had a positive immunodiagnostic result. Locations of previous vacations for this family outside Germany included Spain (not the Balearic Islands) and Corsica, where they bathed frequently in the Cavu River. These epidemiologic findings provide strong circumstantial evidence supporting the presence of a previously unrecognized focus of urinary schistosomiasis in Corsica.

We performed molecular analysis of schistosomal miracidia DNA. The second internal transcribed spacer region of the ribosomal gene complex (*7*,*8*) was amplified and sequenced. Viable eggs obtained from the patients in France were those of *S. haematobium*. Additional molecular investigations are being conducted to assess genetic diversity of this isolate from Corsica and the geographic origin of the introduced parasite.

The malacologic situation in Sainte Lucie de Porto-Vecchio was investigated during May 12–19, 2014; three rivers (Figure) were included in the survey. Four sites were sampled in the Cavu River, and *B. truncatus* snails were found in 3 sites that corresponded to bathing areas (site 1: 41°43′53.57″N, 9°17′36.70″E; site 2: 41°43′22.13″N, 9°17′59.87″E; site 3: 41°42′8.40″N, 9°21′5.82″E). Snails were also found in the nearby Tarcu River (site 5) and Osu River (site 6). These findings confirmed previous data for the presence of *B. truncatus* snails in Corsica (*5**,**6*). Water temperature was recorded at 11:00 am at these 3 sites (range 15°C–16°C). This temperature range is not optimal for the snail intermediate host stage of the parasite life cycle (*9**,**10*). Of 148 live snails that were obtained in the Cavu River, none were infected with schistosome cercariae.

**Figure:**
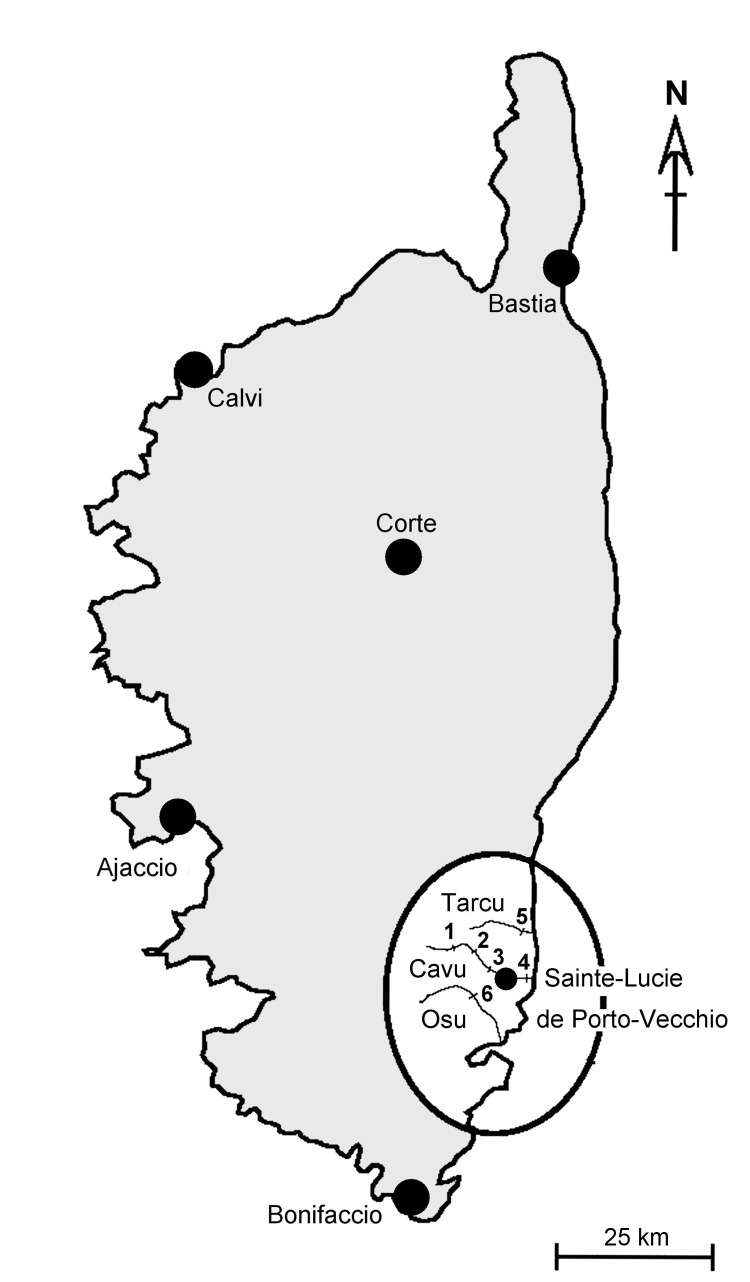
Corsica, France, showing malacologic survey sampling sites (oval) in 3 rivers (Tarcu, Cavu, and Osu). *Bulinus truncatus* snails were found at sites 1, 2, 3, 5, and 6.

Data from the field survey and epidemiologic information for the cases in France and Germany, indicated transmission of schistosomiasis haematobium in the Cavu River in southeastern Corsica in 2011 and 2013. Additional supportive evidence is the fact that the father of the index case-patient had gross hematuria in 2012 and 2013. 

Two hypotheses are proposed to account for this situation. The first hypothesis is that the parasite (i.e., schistosome eggs) was transmitted by an infected person into the Cavu River in June or July 2011, when environmental conditions were favorable for snail infection. However, questions arise about survival of infected snails during the winter and their ability to reinfect the area during the following summers in 2012 and 2013. The second hypothesis is that schistosome eggs were spread by infected persons at the beginning of summer and caused a permanent transmission cycle in this focus. This situation would be difficult to control. Additional information should be obtained by a long-term malacologic survey to detect infected mollusks in this region.
